# S100A7, a Novel Alzheimer's Disease Biomarker with Non-Amyloidogenic α-Secretase Activity Acts via Selective Promotion of ADAM-10

**DOI:** 10.1371/journal.pone.0004183

**Published:** 2009-01-13

**Authors:** Weiping Qin, Lap Ho, Jun Wang, Elaine Peskind, Giulio Maria Pasinetti

**Affiliations:** 1 Department of Psychiatry, Mount Sinai School of Medicine, New York, New York, United States of America; 2 Department of Neuroscience, Mount Sinai School of Medicine, New York, New York, United States of America; 3 Geriatric Research and Clinical Center (GCRC), James J. Peters Veteran Affairs Medical Center, Bronx, New York, United States of America; 4 University of Washington School of Medicine, Seattle, Washington, United States of America; Columbia University, United States of America

## Abstract

Alzheimer's disease (AD) is the most common cause of dementia among older people. At present, there is no cure for the disease and as of now there are no early diagnostic tests for AD. There is an urgency to develop a novel promising biomarker for early diagnosis of AD. Using surface-enhanced laser desorption ionization-mass spectrometry SELDI-(MS) proteomic technology, we identified and purified a novel 11.7-kDa metal- binding protein biomarker whose content is increased in the cerebrospinal fluid (CSF) and in the brain of AD dementia subjects as a function of clinical dementia. Following purification and protein-sequence analysis, we identified and classified this biomarker as S100A7, a protein known to be involved in immune responses. Using an adenoviral-S100A7 expression system, we continued to examine the potential role of S100A7 in AD amyloid neuropathology in *in vitro* model of AD. We found that the expression of exogenous S100A7 in primary cortico-hippocampal neuron cultures derived from Tg2576 transgenic embryos inhibits the generation of β-amyloid (Aβ)_1–42_ and Aβ_1–40_ peptides, coincidental with a selective promotion of “non- amyloidogenic” α-secretase activity via promotion of ADAM (a disintegrin and metalloproteinase)-10. Finally, a selective expression of human S100A7 in the brain of transgenic mice results in significant promotion of α-secretase activity. Our study for the first time suggests that S100A7 may be a novel biomarker of AD dementia and supports the hypothesis that promotion of S100A7 expression in the brain may selectively promote α-secretase activity in the brain of AD precluding the generation of amyloidogenic peptides. If in the future we find that S1000A7 protein content in CSF is sensitive to drug intervention experimentally and eventually in the clinical setting, S100A7 might be developed as novel surrogate index (biomarker) of therapeutic efficacy in the characterization of novel drug agents for the treatment of AD.

## Introduction

Alzheimer's disease (AD) is the most common cause of dementia among people age 65 and older. AD affects approximately 4 million Americans, of which nearly half are in health care institutions [1]. Both neuritic senile plaques (NPs) and neurofibrillary tangles (NFTs) are the pathological hallmarks of the disease, with progressive loss of neurons in the brain. In some vulnerable brain regions, such as certain cortical areas, more than 50% or even up to 70%–90% of neurons are lost [2]. As a result, a diseased brain may show overall shrinkage, severe cortical atrophy, and ventricular enlargement in late stages of the disease [3].

At present, there is no cure for the disease and early diagnosis is all but impossible. The onset of disease is not manifested clinically and little is known regarding the cause of non-familiar AD. There is also no definite clinical method to determine in which patients with mild cognitive impairment progresses to AD with dementia [4]–[5]. Therefore, there is an urgency to develop a novel promising biomarker for early diagnosis of AD [4].

AD often goes unrecognized or is misdiagnosed in its early stages, often because its symptoms are mistaken for inevitable consequences of aging. Even as the disease progresses, clinical diagnosis can be made with only 65%–90% accuracy. A definitive diagnosis of AD can only be made after death, when autopsy can reveal senile plaques and neurofibrillary tangles in brain tissue. The plaques result from aggregation of β-amyloid peptides and were thought to be involved in the pathogenesis of AD; however, their presence does not always correlate with neurologic symptoms [6]. Identification of an AD biomarker for early clinical AD diagnosis is a critical first step in the development of methodologies for early disease intervention, and would have several advantages. It would be able to identify AD at a very early stage of the disease, before the cognitive symptoms are found in neuropsychologic tests, and before brain-imaging studies could reveal degeneration. Differential diagnosis of AD is difficult, and a biomarker reflecting neuropathologic changes at the molecular level in the brain could distinguish AD patients not only from those individuals with mild cognitive impairment who do not develop AD but also from patients with depression. A biomarker would also greatly help screening new therapies, especially those directed to prevent neuropathological changes. Until now, AD cannot be diagnosed by a valid clinical method or a biomarker before the disease has progressed so far that dementia is present. Furthermore, no valid method is available to determine which patient with mild cognitive impairment (MCI) will progress to AD. Therefore, a correct diagnosis in the early stage of AD is not only of importance considering that early drug treatment is more effective but also that the psychological burden of the patients and relatives could be decreased [7].Many ante-mortem biochemical markers for AD have been investigated. Plasma, erythrocytes [8], lymphocytes [9], urine [10], hair [11], and skin [12], have all been analyzed.

Clinical trials of novel therapeutic strategies in AD are disadvantaged by our lack of understanding of the pathophysiology of AD. Because AD cannot be diagnosed until well into the disease cycle, clinical trials with potential novel disease-modifying drugs are conducted in advanced AD dementia cases, which often mean that the disease is too far along for the agents to have much effect. In addition, there is the lack of understanding in the mechanism that supports application of specific drugs and the lack of a rational dose and clinical stage of AD for application of novel drugs to treatment populations. Further characterization of novel indexes of AD clinical progression and pathophysiology may provide insight that might facilitate the development of “customized” therapeutic intervention for specific stages of the disease. Because AD is sometimes undiagnosed or misdiagnosed, it is extremely important to identify robust and easily measured biological markers for the disease, especially markers that detect early or impending disease. Such a panel of markers could allow for early therapeutic treatment intervention to potentially restore memory formation, stop the deposition of insoluble amyloid-beta plaques and prevent neurodegeneration. Biomarkers may also allow real-time testing to monitor whether disease progression has been changed by therapeutic interventions [13].

In this study using proteomic approaches we report for the first time the identification and characterized of a protein S100A7 previously implicated in inflammatory responses and cell differentiation among other functions [26] in the CSF and brain of AD. We sought to understand how S100A7 may influence AD clinical dementia, and found that S100A7 may selectively attenuate AD amyloid neuropathology through promotion of the “non-amyloidogenic” α-secretase processing of APP.

## Materials and Methods

### Protein profiles in AD CSF and serum using SELDI MS

All individuals underwent evaluation that consisted of medical history, physical and neurologic examinations, laboratory tests, and neuropsychological assessments. Control subjects had no signs or symptoms of cognitive decline or neurologic disease including epilepsy; all subjects had a MMSE score between 28 and 30; a CDR score of 0; and NYU paragraph recall scores (immediate and delayed) >6. Lumbar puncture (LP) was performed with a 24G spinal needle. Individuals remained at bed rest for one hour following LP. Only AD patients with pathological confirmation of AD according to NIA-Reagan criteria (high), to the exclusion of Lewy body disease or vascular disease were included in this study. All CSF for proteomic analysis was taken from the 15^th^ to 25^th^ ml collected to limit variations arising from rostral-caudal gradient. In addition, all LP was performed in the morning to limit potential circadian fluctuation of CSF proteins and metabolites. Clear CSF specimens were collected and immediately frozen and stored at −70°C. The protein concentration in CSF is relatively low compared to plasma (CSF:plasma = 1/20–100), and in addition, the protein profiles in CSF are similar to those in plasma, suggesting that even a minor contamination of CSF with blood could significantly confound the interpretation of quantitative proteomic analysis of CSF. To minimize blood contamination in our CSF samples, only CSF samples with <10 RBCs/ml and a serum: CSF ApoB (a protein not generated in CNS) CSF protein expression profiles were analyzed using high-throughput SELDI mass spectrometry. Control samples were always run in parallel with the experimental samples and the signals in the resulting profiles of each chip were simultaneously normalized and represented individually, via integrated software, allowing for direct comparison of individual protein expression profiles. All samples for the proposed study were obtained from Dr. Elaine Peskind at University of Washington (UW). In order to provide protection to research subjects, it is policy at UW to obtain “double consent” from both the subject and their legally authorized representative when the subject is a member of a cognitively impaired group. The standard procedure at UW is that all subjects with cognitive impairment have a legally authorized representative (using Washington statutory definition) co-sign all consents. We attempt to protect each subject's rights and welfare by explaining to both the subject and the person with signing authority that this study is voluntary, they will not lose any other benefits to which they are otherwise entitled by not participating, that there are risks associated with this research, and that if they are not currently taking one of the FDA-approved drugs for treatment of dementia that they may want to consider trying one of these prior to getting involved with this study.

In all cases, we make sure that a subject with cognitive impairment is manifesting assent before beginning or continuing any study procedure (whether it be as minor as taking vital signs or as major as performing an LP). If at any time, a subject with cognitive impairment expresses desire to skip or discontinue a study procedure, we respect that subject's wishes. Because people with cognitive impairment sometimes have difficulty verbally expressing themselves, if there is any indication at any time during the study, verbally or physically, that they do not want to participate, we will discontinue them. The only exception would be that if a subject with cognitive impairment wishes to leave the Clinical Research Unit before it is appropriate (i.e., after the LP, we prefer to have the subject lie in bed for one hour to minimize any potential for side effects). In such a case, we would do our best to have the subject continue study protocol as much as possible.

The Institutional Review Board of the University of Washington approved the use of human subjects for this study.

In this study, human CSF samples were analyzed using two types of ProteinChips: Immobilized Metal Affinity Capture (IMAC) chips and Strong Anion Exchange (SAX) chips, which, respectively, selectively capture divalent metal-binding proteins and anionic (basic) proteins for analysis by “time of flight” mass spectrometry. For SELDI analysis using the IMAC chip, an eight-spot IMAC chip was first activated by incubating twice (15 min each) with copper sulfate (100 mM in water). The chip was then washed with water, followed by washing with sodium acetate (50 mM in water) and a final wash with water. The chip was then assembled with a “Bioprocessor” (Bio-Rad), which allows for applications of large volumes onto each of the ProteinChip spots, and individual CSF samples were immediately applied over each of the spots. In this study, independent CSF specimens containing 1 µg of total protein were adjusted to a final volume of 120 µl PBS/0.1% Triton-X 100 for SELDI analysis. Samples were shaken (250 rpm) for 1 hour at room temperature. The ProteinChip was washed three times (5 min each) with PBS containing 0.1% Triton-X 100 followed by a quick rinse with water. While the chip surface was still moist, 1 µl of saturated sinapinic acid (SPA) solution (in 50% acetonitrile/0.5% trifluoroacetic acid) was applied on each spot. After drying, the profile of divalent metal-binding proteins captured on each of the spot was analyzed by “time of flight” mass spectrometry using a PBS II ProteinChip reader system (Bio-Rad).

For selective capturing and expression-profile analysis of anionic protein species from CSF, 2 µg of total protein from individual specimen was adjusted to a final volume of 200 µl with binding buffer [20 µM Trisma base (pH 8) containing 0.1% Triton-X 100]. A SAX ProteinChip was assembled with a “Bioprocessor” and each spot was prewashed (shaking at 250 rpm for 5 min at room temperature) with 350 ml of 20 mM Trisma base (pH 8) containing 5 M NaCl and 0.1% Triton-X 100. Thereafter, the prewash solution is exchanged with CSF samples following by incubation (shaking at 250 rpm at room temperature) for 1 hour. The chip was washed 3 times (5 min each) with binding buffer followed by a brief rinse with water. SPA (1 µl) was applied to each spot and anionic proteins captured on to each of the spots were analyzed using the PBS II system. In all SELDI “time of flight” mass spectrometry analysis using either the IMAC or the SAX ProteinChip, individual protein-expression profile spectra were collected using an average of 80 laser shots (with the laser intensity set at 180, and detector sensitivity set at 6 on the PBS II system). Spectra collected were analyzed using the ProteinChip software version 2.1c, which highlights differences in expressions of specific protein species [defined by mass (Da)/charge] between treatment groups (e.g., CSF from animals treated with FCA vs. animals treated with FCA plus nimesulide).

### ELISA measurement of S100A7 protein in CSF

CSF samples were coated in Nunc-Immuno plates (Maxisorp, Nunc, Roskilde, Denmark) in 100 µl of Buffer 42 (30 mM NaHCO_3_, 70 mM Na_2_CO_3_, 0.05% NaN_3_, pH 9.6) at 4°C for O/N. The antigen from the wells was aspirated and tapped on absorbent paper to remove excess liquid. It was washed well with 400 µl PBS, incubated for 15–30 seconds, aspirated, and then repeated once. The plate was blocked with 300 µl of blocking solution (TBS+0.1%Tween+5%milk) and incubated for 1–2 hours at room temperature with rocking. Blocking solution was removed and incubated with S100A7 monoclonal antibody (1∶500) (Imgenex, San Diego, CA) for 2 hours. The microplates were washed 4 times with 400 µl per well of PBST. Wells were incubated with 100 µl of diluted horseradish peroxidase-conjugated mouse IgG (1∶2000) at room temperature for 2 hours. The microplates were washed 4 times with 400 µl per well of PBST. TMB [Microwell Peroxidase Substrate System, Kirkegaard & Perry Laboratories, Maryland, USA] were added (100 µl). Plates were allowed to develop until the second to the least concentration standard had a slight color change; the reaction was then stopped by adding 100 µl 1.8 N H_2_SO4 (10% H_2_SO4 or 5.7% O-phosphoric acid) to each well. The plates were read plates at 450 nm within 30 min of adding the solution.

### Postmortem AD brain

Human postmortem brain samples from AD and age-matched non-AD cases were obtained from the Alzheimer's Disease Brain Bank of the Mount Sinai School of Medicine. The cases selected had either no significant neuropathological features or only neuropathological features associated with AD. A multistep approach based on cognitive and functional status during the last 6 months of life was applied to the assignment of clinical dementia rating (CDR). Samples of OC and EC were divided into groups on the basis of their CDRs as follows (OC/EC): CDR 0, non-demented (n = 9/9); CDR 0.5, at high risk of developing AD dementia (n = 14/9); and CDR 5, severe dementia (n = 9/9).

The extent of amyloid neuritic plaques (NPs) and neurofibrillar tangles (NFTs) staining in the EC-BM36/38 was assessed in accordance with the Consortium to Establish a Registry for Alzheimer's Disease (CERAD) neuropathologic battery [14]. Multiple (∼5) high-power (×200, 0.5-mm^2^) fields were examined in each histologic slide from multiple regions of tissue, according to the CERAD regional sampling scheme. The density of NPs and NFTs was rated on a 4-point scale as follows: 0, absent; 1, sparse; 3, moderate; and 5, severe. Plaques were visualized after either Bielschowsky silver or thioflavin-S staining. Three of the investigators were masked to the clinical diagnosis of each patient until all quantitative analyses were complete and values were assigned to each specimen.

### Detection of human S100A7 mRNA expression by RT-PCR assay

Quantitative human S100A7 real-time (RT)-PCR was conducted as previously described, with minor modifications [15]. Briefly, total RNA was extracted from frozen brain tissue samples (Qiagen, Valencia, CA) and transcribed into cDNA by 40 rounds of PCR amplification in the presence of SYBR Green I (Molecular Probes, Eugene, OR), human S100A7 primers (forward primer: 5′-AGC CTC AAA CTC CAA ACA CC-3′ and reverse primer: 5′-CAA CAG GGA TTT GAC CAC-3′); using an ABI 7900HT thermal cycler (Applied Biosystems, Foster City, CA) at 95°C for 15 sec (denaturation), 55°C for 15 sec (annealing), and 72°C for 30 sec (extension).

Amplicon size and reaction specificity was confirmed by agarose gel electrophoresis. CTGF RT-PCR amplification was monitored in real time following each thermal cycle by measurements of fluorescent emission generated by incorporation of SYBR Green into double-stranded DNA. Data were transformed into a semi-log plot where the X-axis represents PCR cycle number and the Y-axis represents the log value of fluorescent intensity. For each reaction, the threshold cycle (C_T_), which is the number of PCR cycles corresponding to the detection threshold, was obtained. The copy number of the targeted gene in each sample was interpolated from its median C_T_ values using a standard curve derived from parallel quantitative RT-PCR amplification of purified CTGF PCR product.

### Generation of human (h) S100A7 adenoviruses

To generate human h-S100A7 adenoviruses, h-S100A7 cDNA construct was introduced to the Adeno-X™ genome for generation of recombinant Adeno-X virus, according to the Adeno- X™ expression system manual (Clontech, Palo Alto, CA). Briefly, the full-length S100A7 cDNA was subcloned into the pShuttle vector cassette via XbaI and KpnI. Both pShuttle/h-S100A7 were then transferred into the Adeno-X viral DNA via I-CeuI and PI-CseI sites; the identity of the h-S100A7 -Adeno-X viral DNA was confirmed by nucleotide sequencing. The recombinant viruses were then packaged by transfecting PacI linearized recombinant viral DNA into human embryonic kidney (HEK) 293 cells with the aid of Lipofectamine (Gibco, Rockville, MD). h-S100A7 Adeno-X viral titer was determined by the tissue-culture infectious dose 50 (TCID_50_) method [16]. This identical strategy was used to generate recombinant LacZ-adenovirus (Clontech, Palo Alto, CA) expressing the bacterial β-galactosidase gene (Clontech, Palo Alto, CA), which served as a negative control. All protocols for viral studies were approved by the Mount Sinai Institutional Review Board.

### Cell cultures and treatments

Chinese Hamster Ovary (CHO) cells expressing human amyloid precursor protein (APP) carrying the K670N, M671L Swedish mutation (APP_swe_) (CHO-APP_swe_; gift from Dr. Robakis) were grown in McCoy's 5A medium supplemented with 10% fetal bovine serum, 1% streptomycin/penicillin (GIBCO), and 400 µg/ml G418 (Life Technologies, Carlsbad, CA). For viral infection, CHO-APP_swe_ was seeded at 4×10^4^ cells/cm^2^ and cultured at 37°C in the presence of 5% CO_2_. Following 24 hours of incubation, cultures (50% confluence) were infected with recombinant h-S100A7 or LacZ adenovirus with doses of virus defined as 10 multiplicities of infection (MOI) [17]. Conditioned medium was collected 24 or 48 hours postinfection for amyloid-beta detection. Cell viability was assessed by 3-(4,5-dimethylthiazol-2-yl)-2,5-diphenyltetrazolium bromide (MTT) colorimetric assay of the cell lysate, as previously described [18].

### Primary neuronal cultures

Embryonic (E14) cortico-hippocampal primary neuronal cultures derived from heterozygous Tg2576 transgenic mice were prepared as previously described [19]. Briefly, after brain dissection, mechanical trituration, and centrifugation, neurons were seeded onto poly-D-lysine–coated 12-well plates at a density of 1×10^6^ cells per well and cultured in neurobasal/B27 medium. The absence of astrocytes (<1–2%) was confirmed by the lack of glial fibrillary acidic protein (GFAP) immunostaining verified in parallel studies (data not shown).

All mice protocols were approved by the Mount Sinai Institutional Animal Care and Use Committee.

### S100A7, sAPPα, sAPP total, holo APP, ADAM-10, ADAM-9, TACE (ADAM-17), MAPK, and PKC immunodetection

Following adenoviral infection and/or incubation with drugs for the appointed time, conditioned media was collected and tissue cultures lysed in RIPA buffer (1×PBS, 1% NP-40, 0.5% sodium deoxycholate, 0.1% SDS, and 0.1 mM EDTA) in the presence of a protease-inhibitor cocktail (Sigma, St Louis, MO) on ice, and stored at −20°C. For immunoblot analysis, protein content was determined by Bradford method (Bio-Rad, Hercules CA), samples were boiled, centrifuged and proteins resolved electrophoretically by SDS-PAGE (10%). Proteins were transferred to nylon transblot membranes (Bio-Rad) and immunoreacted with appropriate antibody. In these studies immunoreactivities were visualized by fluorescence autoradiography using ECL chemiluminescence detection (SuperSignal Chemiluminescent Detection Kit, Pierce Biotechnology, Rockford IL).

Monoclonal S100A7 was used at 1∶500 dilution (Imgenex, San Diego, CA). Polyclonal C8 antibody (1∶1,000 dilution, raised against amino acids 676–695 of human APP, a gift from Dr. Selkoe) was used for detection of total human holo-APP. Monoclonal 22C11 antibody (1∶1,000 dilution, Chemicon International, Temecula, CA) recognizing amino acids 60–100 of an N-terminal epitope of human APP was used to quantify total soluble (s)APP. Monoclonal 6E10 antibody (1∶1,000 dilution, Senetek, St. Louis, MO) recognizing amino acids 1–17 of the β-amyloid domain of APP (a site that constitutes the C terminus of sAPP peptide) was used to quantify the level of sAPPα. Polyclonal ADAM-10 (1∶500 dilution, Chemicon, CA), polyclonal ADAM9 antibody (1∶500 dilution, Chemicon International, Temecula, CA), polyclonal TACE antibody (1∶1,000 dilution, Santa Cruz Biotechnology, CA), polyclonal BACE (M-83) antibody (1∶200 dilution, Santa Cruz Biotechnology, CA), and polyclonal anti-IDE antibodies (clone BC2 1∶500 dilutions, gift from Dr. Eduardo Castano) were used for detection of the levels of ADAM-10, ADAM-9, TACE (ADAM-17), BACE and IDE, respectively. In these studies β-actin immunoreactivity (1∶3,000 dilution, Sigma, MO) controlled for selectivity of changes.

### Measurement of α-secretase and β-secretase activity

α-secretase and β-secretase activities were measured using commercially available kits (R & D Systems, Minneapolis, MN). Briefly, cultured cells or brain samples were homogenized in supplied buffers. The homogenate containing 200 µg total protein was then added to alpha-secretase–specific APP peptide (YEVHHQKLV for alpha-secretase activity assay and REEVNLDAEFKR for beta-secretase activity assay) conjugated to the reporter molecules EDANS and DABCYL. In the uncleaved form, the fluorescent emissions from EDANS are quenched by the physical proximity of the DABCYL moiety, which exhibits maximal absorption at the same wavelength (495 nm). Cleavage of the peptide by the secretase physically separates the EDANS and DABCYL, allowing for the release of a fluorescent signal. This signal may be quantitated at an excitation/emission wavelength of 355/510 nm. The level of secretase enzymatic activity in the homogenate is proportional to the fluorometric reaction (R&D Systems).

### CTF-γ assay

In h-S100A7 or LacZ-infected cells, levels of CTF-γ cleavage product of APP were assessed from membrane preparations, as previously described [20]–[23]. Briefly, cell monolayers were rinsed twice with ice-cold PBS on ice, scraped from tissue-culture dishes and centrifuged (1,500 rpm, 10 min, 4°C). Cell pellets were then resuspended (0.5 ml/10 cm dish) in homogenization buffer [10 mM MOPS, pH 7.0, 10 mM potassium chloride, 1× complete protease inhibitor (PI)] (Roche Molecular Biochemicals, Germany), and homogenized by passing cell suspensions through a 23-gauge needle 10 times. Homogenates were then centrifuged (2,500 rpm, 15 min at 4°C) to remove unbroken cells and nuclei. The supernatant (membrane and postnuclear supernatant) was then centrifuged (14,000 rpm, 20 min, 4°C) and rinsed in homogenization buffer. Membranes were then resuspended in assay buffer (150 mM sodium citrate pH 6.4; 1× PI) and incubated (2 hr) at 37°C in 25 µl of incubation buffer per assay sample to allow for generation of the CTF-γ cleavage product; negative control samples were maintained on ice. CTF- γ cleavage products (as well as CTF-α and CTF-beta) were resolved electrophoretically in 10–20% Tris-Tricine gels (Bio-Rad, CA), and identified using the anti APP polyclonal C8 antibody. Immunoreactivities were visualized autoradiographically using a chemiluminescence detection kit (SuperSignal; Pierce Biotechnology, Rockford, IL).

### Aβ ELISA assays

For detection of β-amyloid peptide generation, conditioned media from CHO-APP_swe_ cells were centrifuged (3,500×g, 10 min, 4°C) to remove cellular debris. β-amyloid_1–40_ or β-amyloid_1–42_ was assessed by sandwich ELISA, according to manufacturer's instructions (BioSource, Camarillo, CA).

### S100A7 cell-surface receptor assessment

For the study exploring the presence of cell-surface S100A7 receptors we used an alkaline phosphatase (AP)–S100A7 fusion protein as a ligand. To generate the AP–PIF fusion protein, a cDNA encoding the entire mature human S100A7 protein was introduced into the 3′-poly-linker site of the AP-TAG-5 expression vector (GenHunter, Nashville, TN), downstream of the AP-coding domain. This recombinant AP-S100A7 construct was transiently transfected into HEH 293 with the aid of Lipofectamine (Life Technologies, Rockville, MD). The AP-S100A7 construct directs the expression of an N-terminal alkaline phosphatase (AP) C-terminal S100A7 fusion protein, which is secreted into the culture medium. Condition medium was collected 3 days after transfection for *in situ* staining of S100A7 receptor. Comparable strategies will be used to generate AP as a negative control for *in situ* staining studies. The AP-S100A7 fusion protein was used to identify S100A7-receptor in mouse brain, primary mouse neuron culture, CHO-APP_swe_ cells, and platelets as described [24]–[25]. For the frozen brain-tissue sections, specimens were cryosectioned into 10 µm-thick sections, which were then mounted onto SuperFrosted-plus slide (Fisher). In preparation for *in situ* S100A7 receptor staining, tissue sections or cultured cells or platelets were fixed for 1 hour in 4% paraformaldehyde at room temperature. After washing for 5 min in HBS (150 mM NaCl, 20 mM HEPES, pH 7) followed by rinses (2×5 min) in HBAH (Complete 1× Hanks Balanced Salt Solution, 0.5 mg/ml BSA, 20 mM HEPES, pH7), the tissue section was incubated with conditioned media containing AP-PIF fusion protein at room temperature for 90 minutes. In parallel control experiments, adjacent tissues or cell sections were incubated with conditioned media containing AP protein. Thereafter, tissue or cell sections were washed in HBAH (6×5 min) and fixed in acetone-formalin (65% acetone, 8% formalin, 20 mM HEPES, pH 7) for 15 sec, and washed again in HBS (2×5 min). Tissue sections or cell sections were then incubated in HBS for 20 min at 65°C. After washing in AP staining buffer (100 mM NaCl, 5 mM MgCl, 100 mM Tris, pH 9.5 made fresh before use), S100A7-receptor interaction was visualized based on development of AP activity. Following brief fixing (8% formalin in PBS for 20 min) and washing (PBS+10 mM EDTA), AP-h-S100A7 stained tissue sections were mounted with aqueous mounting medium and stored in the dark at room temperature. Immunoreactivities were visualized using the ABC method (Vector).

### Preparation of platelet

Human platelet was prepared from blood collected from a cognitively normal control case. Freshly collected blood was centrifuged at low speed (200×g) for 15 min at room temperature and resulted in platelet-rich plasma (PRP). For analysis of cell-surface receptor in platelet, 200 µl of the recovered PRP was plated in 8-well chamber slides, and left overnight. The supernatant was then removed, and the attached platelet treated with 100 µl AP or AP-S100A7 expressing culture medium for 24 hours. For analysis of α-secretase activity and ADAM-10 levels in platelet, 200 µl PRP was collected in 1.5 ml microcentrifuge tube and treated with 100 nM His-S100A7 recombinant protein (gift from Nelly Polyak, Harvard Medical School) for 24 hours. Platelets were collected by centrifugation at 3,000×g for 15 min at 0°C. The resulting platelets were subjected to α-secretase activity and ADAM-10 measurement as described above. This study was approved by the Institutional Review Board at James J. Peters Veterans Affair Medical Center approved.

### Generation of double transgenic h-S100A7 mice

h-S100A7 transgenic mice were of the C57B6/SJL background. The expression of h-S100A7 transgene in transgenic mice is driven by the cytomegalovirus immediate-early enhancer linked to the chicken beta-actin promoter. Western blot analysis of serum collected from h-S100A7 transgenics showed significant higher levels of immunodetectable S100A7, compared to serum collected from age-, gender- and strain-matched wild-type control mice.

All mice protocols were approved by the Mount Sinai Institutional Animal Care and Use Committee.

### Statistical analysis

All values are expressed as mean±standard error of the mean (SEM). Differences between means were analyzed using a two-tailed Student *t* test while difference among means were assessed ANOVA. In all analyses, the null hypothesis was rejected at the 0.05 level. All statistical analyses were performed using the Prism Stat program (GraphPad Software, San Diego, CA).

## Results

### Identification of S100A7 as an AD biomarker

**Figure 1:**
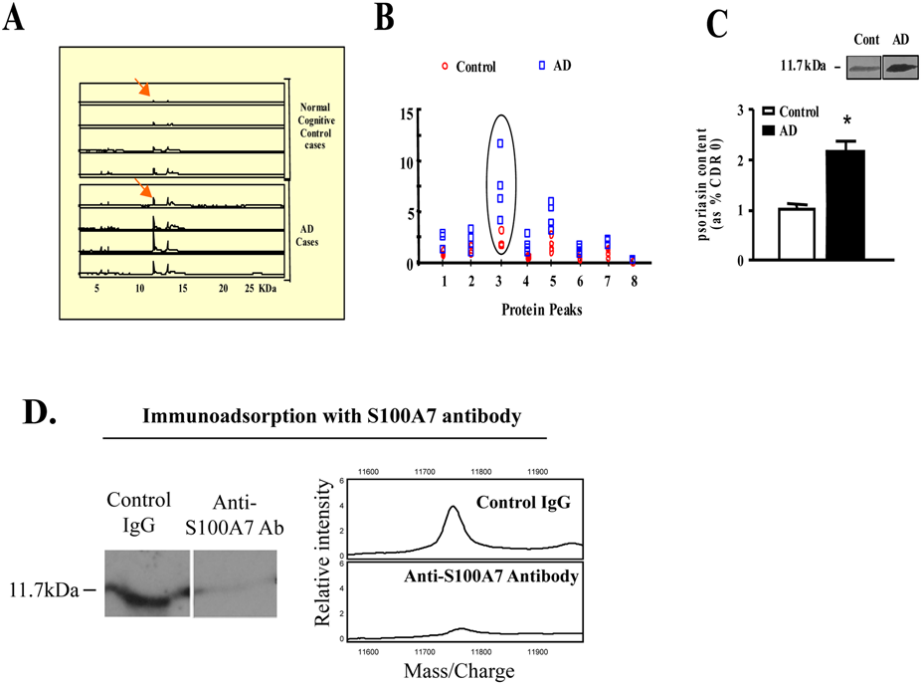
Identification of biomarkers in the CSF of AD cases. For the study, CSF was collected by lumbar puncture (n = 4 per group), centrifuged (1,300×g) at 4°C, aliquoted and stored at −80°C. 4 µg of CSF proteins from each case was analyzed by SELDI technology using the IMAC-Cu^++^ protein chip; only Cu^++^-binding proteins are analyzed using this chip. *A*, SELDI retention map; “peaks” represent individual detected proteins, and the area under the peak represents the signal intensity. Reference molecular sizes are indicated across the bottom of the panel. *B*, molecular weight frequency scatter graph indicating the quantitative distributions of individual Cu^++^-binding protein for each of the cases analyzed. Peak #3 shows the levels of expression of the 11.7-kDa species in the AD dementia and normal cognitive controls. *C*, western blot confirmation of elevated S100A7 contents in the CSF of AD cases. Bar graph represents mean±SEM and is shown as % of the control group; *P<0.05, by 2 way t-test, n = 4–7 per group. Inset: representative S100A7 western blot analysis. D. Immunoadsorption with the S100A7 antibody showed no visible bands in a western blot.

In SELDI proteomic studies exploring differentially expressed protein in the CSF we identified an 11.7-kDa metal-binding protein whose content which is elevated in the CSF of mild (CDR1) AD cases (Fig. 1A, B), relative to normally cognitive controls. Based on this evidence the 11.7 kDa protein species was isolated from a pool of CSF from AD dementia cases (CDR 2; moderate AD dementia) and sequence identified as S100A7 based on MS criteria.

Control immunoadsorption studies using MS-SELDI technology confirmed the SELDI-MS evidence and demonstrated that S100A7 content is elevated in the CSF of moderate AD dementia cases (CDR2) relative to the normal controls (Fig. 1C, D).

**Figure 2:**
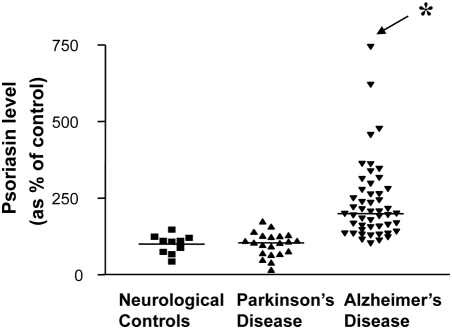
Selective elevation of S100A7 protein in the CSF of AD cases relative to neurologically normal control cases or PD cases. Monoclonal anti-human S100A7 antibody was used in a single antibody ELISA assay to quantify the content of S100A7 immunoreactivity in the CSF of non-medicated AD (n = 49), Parkinson's disease (n = 21), and neurological normal control (n = 10) cases. In this study, microtiter wells were coated with the antigen (0.5 µl CSF diluted 200-fold), dissolved in 100 µl of Buffer 42 (30 mM NaHCO_3_, 70 mM Na_2_CO_3_, 0.05% NaN_3_, pH 9.6), and incubated at 4°C overnight. Data are shown as a scatter plot. *P<0.05 vs. neurological controls or PD cases by one-way ANOVA.

Using an established S1000A7 ELISA assay in the lab we confirmed that the increased steady-state levels of S100A7 immunoreactive material in the CSF of AD cases (CDR 2–5) was rather selective since no detectable changes were found in CSF of cases affected by Parkinson's disease without dementia and normal neurological control cases (Fig. 2)

### S100A7 mRNA expression is regulated in the brain as a function of AD clinical progression and amyloid neuropathology

Based on the evidence that S100A7 protein species in CSF might be an AD biomarker, we continued to explore the expression of S100A7 in the AD brain and tested the hypothesis that S100A7 expression may be regulated as a function of clinical dementia or neuropathology.

**Figure 3:**
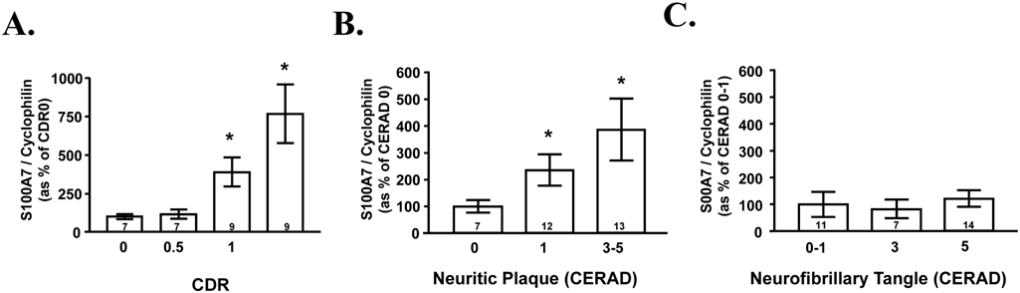
S100A7 mRNA expression in AD brain increases as a function AD dementia and amyloid neuropathology. *A*, S100A7 content in BM 8 (quantified by real-time RT-PCR and normalized by cyclophilin) as a function of CDR representing cognitive normalcy (CDR 0), questionable dementia (CDR 0.5), mild dementia (CDR 1), and severe dementia (CDR 5). Data are expressed as mean±SEM and are shown as percent of CDR 0 group. *B*, *C*, S100A7 mRNA expression as a function of β-amyloid NP plaque and NFTs neuropathology, respectively, in accordance with the CERAD four-point scale for AD. Data represent mean±SEM and are shown as percent relative to CERAD 0 group, respectively; in A–C statistics were calculated by two-way ANOVA followed by Dunnett's t-test vs. control; *P<0.05. The number inside each bar indicates number of cases analyzed.

We found that S100A7 mRNA expression increases in the brain (frontal cortex; BM8) as a function of AD dementia assessed by CDR (Fig. 3A). Most importantly, we found that S100A7 mRNA expression selectively correlated with AD amyloid plaque (Fig. 3B), but not neurofibrillar tangle as assessed by CERAD neuropathology score (Fig. 3C). These changes in S100A7 expression in the frontal cortex of the brain were highly selective because no detectable changes were visualized in the occipital cortex (BM17, data not shown).

### S100A7 is involved in the “non-amyloidogenic” α-secretase activity in AD

**Figure 4:**
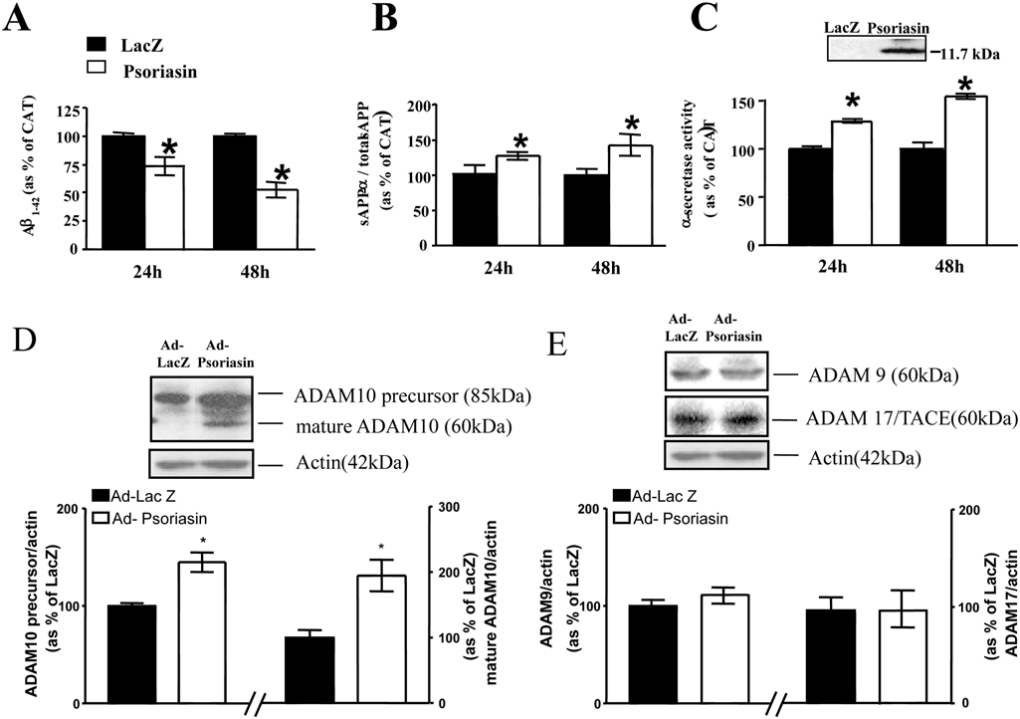
S100A7 expression attenuates β-amyloid peptide generation in cultured CHO-APP_swe_ cells. Cells were infected with adeno human h-S100A7 virus or lacZ virus for 24 or 48 hours at 10 multiplicities of infection (MOI = 10). *A*, Amyloid-beta_1–42_ peptide content in the condition media was analyzed using a commercial ELISA assay. *B*, western blot analysis of soluble (s)APPα (6E10 antibody/total sAPP content (22C11 antibody) in the culture media. *C*, α-secretase activity of cell lysate assessed based on monitoring cleavage of a specific synthetic α-secretase peptide substrate using an α-secretase activity kit following manufacturer's instructions (R&D Systems). *Inset*, representative western blot analysis of h-S100A7 in culture media confirming the secretory feature of S100A7; Mature ADAM-10 (*D*) and mature ADAM-9 or ADAM-17 (TACE) (*E*) were assessed by western blot analysis. *Insets*, representative western blot analysis of precursor and mature ADAM-10 (*D*) and ADAM-9 or ADAM-17 (*E*). *A–C*, values represent mean±SEM and are shown as percent of LacZ control infected cells; n = 3 independent cultures per group from two independent studies; *P<0.05 by two-way t-test.

Since S100A7 expression is regulated in the AD brain as a function of clinical dementia as well as amyloid-plaque neuropathology, we hypothesized that S100A7 might play an important role in amyloidogenesis eventually influencing cognitive functions. We found that the adenoviral expression human h-S100A7 in cultured Chinese hamster ovary (CHO) cells (see inset Fig. 4C), stably expressing mutant APP_swe_, significantly attenuated the content of amyloidogenic Aβ_1–42_ peptides in the conditioned medium (Fig. 4A) which coincide with a commensurate elevation of the content of soluble (s)APPα relative to total sAPP (sAPPα/total sAPP) in the conditioned medium, which is an indication of “non-amyloidogenic” α-secretase activity (Fig. 4B). Consistent with this evidence, using a commercially available α-secretase activity assay,^27^ we confirmed that exogenous h-S100A7 expression in CHO-APP_swe_ promotes α-secretase activity, based on quantification of fluorescent signal from specific α-secretase cleaved substrates *in vitro* (Fig. 4C).

In light of recent evidence indicating that the proteinase ADAM-10 (a disintegrin and metalloproteinase) may act as an α-secretase [28], we continued to explore the potential role of S100A7 in the regulation of ADAM-10 in cultured CHO-APP_swe_ cells. We found that exogenous hS100A7-mediated “non-amyloidogenic” α-secretase activity coincided with a selective >two fold accumulation of cellular ADAM-10 precursor protein (85 kDa) and its processed mature form of ADAM-10 (60 kDa), as assessed by western blot assay (Fig. 4D).

The 62-kDa mature ADAM-10 protein species is known to act as an α-secretase *in vitro* and to cleave Aβ-derived peptides at leucine_16_ [29]. No detectable change in ADAM-9 and ADAM-17 (TACE) expression was found in S100A7 expressing CHO-APP_swe_ cells (Fig. 4E). In control studies we found that β-secretase activity (assessed by commercial assay) [27] or γ-secretase activity (assessed by quantitation of γ-CTF generation) [23] are not affected by h-S100A7 expression in CHO-APP_swe_ cells (data not shown).

### Exogenous expression of human S100A7 reduced generation of Aβ peptides in primary neuron cultures from Tg2576 mice, coincidental to induction of α-secretase activity

**Figure 5:**
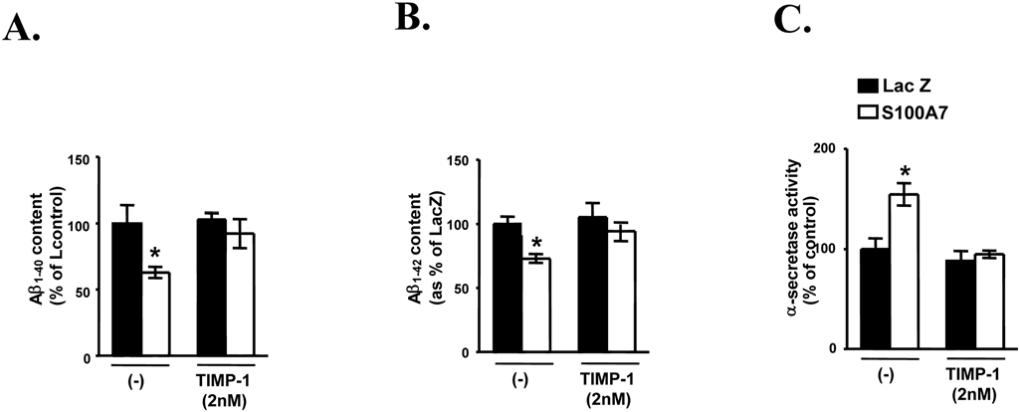
Adenovirus-mediated overexpression of hr-S100A7 modulated β- amyloid generation in primary Tg2576 cortical neurons cultures. Primary cortical neuronal cultures generated from hemizygote E14 Tg2576 mouse embryos were maintained in a serum-free Neurobasal media in the presence of B27-supplement (GIBCO) essentially as previously described [42]. Seven-day-old Tg2576 neuronal cultures were infected with adeno-S100A7 virus (or adeno-LacZ virus as control) with a dose of virus at 10 MOI. (A) Aβ_1–40_ and Aβ_1–42_ (*B*) peptide contents in the condition media 48 hrs after viral infection was analyzed using commercial ELISA assays. *C*, α-secretase activity of neuronal cell lysate using the α-secretase activity kit (R&D Systems).

Consistent with the observations in CHO-APP_swe_ cells, using activity assays as discussed above we confirmed that viral expression of exogenous h-S100A7 expression in primary neuron cultures derived from Tg2576 embryos promotes cellular α-secretase activity coincidental with significant attenuation of accumulation Aβ_1–40_ and Aβ_1–42_ peptide content in the conditioned medium 48 hrs after infection, relative to Lac-Z infected neurons (Fig. 5A–C). Interestingly the h-S100A7 mediated induction of α-secretase activity coincidental with attenuation of Aβ peptide accumulation in the conditioned medium is prevented by co-treatment of cortico-hippocampal neurons with the ADAM-10 inhibitor, TIMP-1 (Fig. 5A–C) further supporting the hypothesis that the S100A7-mediated attenuation of Aβ accumulation in the cortico-hippocampal neurons is ADAM-10 dependent.

In further control studies we found that the h-S100A7 overexpression in cortico-hippocampal neurons derived from Tg2576 embryos did not affect the cellular contents of either the precursor or the mature (catalytically active) ADAM-17 another members of the ADAM proteinase family ADAM-17 (TACE), which can also act as α-secretase in certain conditions [29], [31] (Data not shown).

### S100A7 influences α-secretase and β-amyloid steady-state levels through mechanisms that involve p42/44 MAPK (Erk1/2) and PKC signaling in primary cortico-hippocampal neuron cultures

There is evidence that α-secretase–associated sAPPα secretion is regulated in a PKC-dependent or -independent fashion that involves activation of tyrosine kinase [32]. Moreover, the MAPK signaling pathway was recently implicated in both PKC and tyrosine kinase receptor regulation of APP processing [33], [34]. We therefore explored which, if any, of these kinases is involved in mediating the action of S100A7 on sAPPα release.

**Figure 6:**
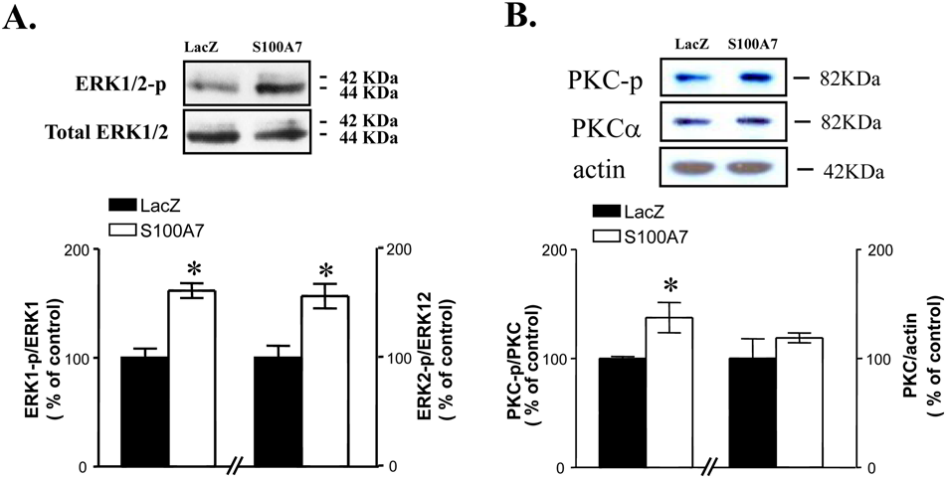
h-S100A7 adenoviral infection in primary cortico-hippocampal neuron cultures derived from Tg 2576 embryos coincides with activation of p42/44MAPK and PKC signaling. *A,B*, cortico-hippocampal neuron cultures derived from Tg2576 embryos were infected with LacZ or h-S100A7 adenovirus for 24 hours at 10 MOI. The phosphorylation of p42/44 MAPK and PKC were analyzed in cell lysate and detected by pTpY^185/187^-p42/44 MAPK antibody or total p42/44 MAPK antibody (*A*) and phosphor-PKC (pan) antibody or PKCα antibody (*B*). In this study, tissue-culture dishes were placed on ice (4°C) and, following the removal of conditioned media, cells were immediately collected in ice-cold 1× SDS-sample buffer containing protease inhibitors. Data represent mean±SEM of determinations made in three separate cultures; *n* = 3 to 4 per culture; two-tailed Dunnett's t-test vs. LacZ infection: *P<0.05, two-way t-test.

We found that, coincidental with the increasing level of α-secretase activity secretion as well as the reduction of Aβ_1–42_ level as shown in primary cortico-hippocampal neuron cultures (see Fig. 5), exogenous expression of h-S100A7 promoted Erk1/2 (Fig. 6A) and PKC-p (Fig. 6B) phosphorylation relative to controls LacZ expressing cells, assessed by western-blotting analysis using a anti-phospho-p42/p44 MAPK or anti phosphor-PKC (pan) antibody. No detectable change in total p42/44MAPK, total PKCα (or control actin) concentrations were found after h-S100A7 expression (Fig. 6A, B).

To further investigate the observation that S100A7 influences α-secretase activity and Aβ steady-state levels in the conditioned medium in cortico-hippocampal neuron cultures through mechanisms that involve p42/44 MAPK (Erk1/2) and PKC-signaling pathways, neuron cultures were pre-incubated for 30 min with PD98059 (10 µM) or GF109203X (5 µM), which are select inhibitors of p42/44 MAPK or PKC, respectively, and then infected cells with adenoviral h-S100A7 or adenoviral lacZ for 24 hours.

**Figure 7:**
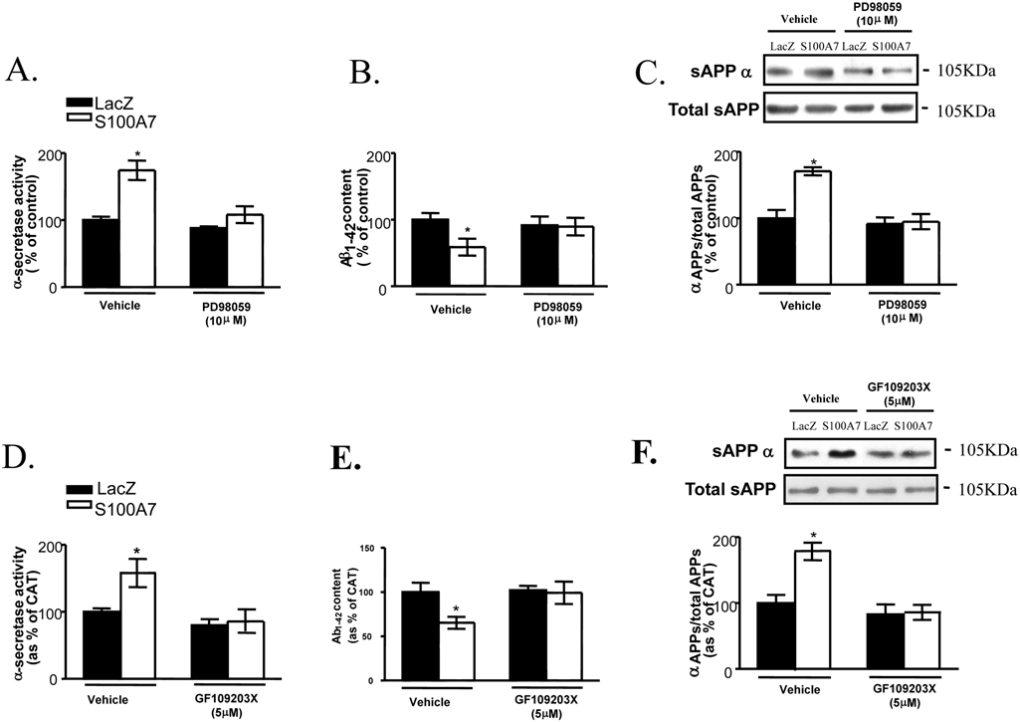
h-S100A7-mediated promotion of α-secretase activity and sAPPα secretion and decreased amyloid-beta_1–42_ level are prevented by p42/44MAPK and PKC signal-transduction inhibitors. In this study cortico-hippocampal neuron cultures derived from Tg2576 embryos were pre-incubated for 30 min with vehicle alone or with PD98059 (10 µM) (*A–C*) or with GF109203X (5 µM) (*D–F*). Following the preincubation time, the cells were infected with LacZ or h-S100A7 adenovirus for 24 h at 10 MOI. *A*, resulting cell lysates were analyzed for α-secretase activity. *B*, *C*, proteins released into the conditioned media were collected and analyzed for β-amyloid by ELISA or sAPP α and total sAPP by western blotting. Data represent mean±SEM of determinations made in two separate cultures; *n* = 3 to per culture; two-tailed Dunnett's t-test vs. control (vehicle treated): *P<0.05.

We found both PD98059 and GF109203X prevented hS100A7–mediated changes in α-secretase activity (Fig. 7A, D) in cell lysates, Aβ_1–42_ steady-state levels in the conditioned medium (Fig. 7B, E), and sAPPα secretion in conditioned media (Fig. 7C, F), relative to the Lac-Z expressing control neuron cultures. These findings indicate that PKC- and MAPK-dependent signaling pathways are involved in S100A7-mediated stimulation of α-secretase activity and reduction of Aβ amylodosis.

### Detection of cell-surface S100A7 receptor(s) in neuronal cells, cultured CHO-APP_swe_ cells, and mouse brain

S100A7 is known to be both a cytoplasmic and but also a secreted protein in certain peripheral cells [26]. Based on this consideration and on our observations, we hypothesized that S100A7 in the brain and in biological fluids, e.g. CSF may promote “non-amyloidogenic” α-secretase activities through an autocrine/paracrine function. To test this hypothesis and to provide supportive evidence for promotion of “non-amyloidogenic” α-secreatse role of S100A7 directly in neurons, we examined a potential interaction of S100A7 with cell-surface receptor(s) in Tg2576 neuronal cultures.

**Figure 8:**
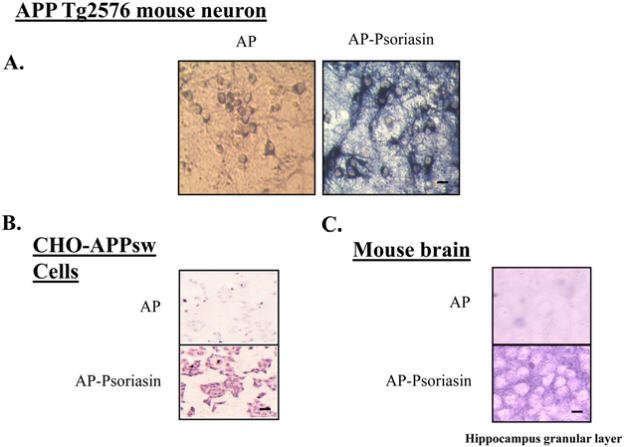
In situ S100A7 receptor binding activities in primary embryonic cortical neurons derived from Tg2576 mice, CHO-APPswe cells, and mouse brain. Seven-day-old Tg2576 neuronal cultures (*A*), CHO-APPswe cells (*B*) and 10 µm frozen tissue sections from mouse brain (*C*) were incubated with AP-S100A7 fusion protein or control AP protein. Blue (*A*) and purple (*B*, *C*) staining detects interaction between AP-S100A7 with a putative S100A7 receptor. Length bar = 10 µm. n = 3 independent cultures per group from two independent experiments.

Using an established N-terminal alkaline phosphate (AP) C-terminal S100A7 fusion protein as a ligand for *in situ* receptor-binding assays [25], we found strong AP-S100A7 binding activity in *in vitro* cortico-hippocampal neuron cultures primarily localized to neuropil and neuronal cell contours supporting for the first time the expression of cell-surface S100A7 receptor(s) in neuronal cells. Control cultures treated with AP alone resulted in background staining, as expected (Fig. 8A).

As expected, cell-surface S100A7 binding activity was detected in cultured CHO-APP_swe_ cells (Fig. 8B) as well as and in the granular layer of mouse hippocampal formation with a preferential distribution with the neuropil (Fig. 8C); AP treated samples resulted in background staining, as expected (Fig. 8B.C).

The study support the hypothesis that secreted S100A7 in the brain, by interaction with cell-surface receptor(s), might promote activation of ADAM-10, ultimately resulting in the promotion of “non-amyloidogenic” α-secretase activity in neuronal cells.

### Exogenous S100A7 expression in platelets promotes “non-amyloidogenic” α-secretase activity

A recent study documented significantly reduced levels of ADAM-10 in circulating blood platelet cells of AD patients compared to age-matched control cases [35]. Although the mechanisms leading to reduction of ADAM-10 in circulating platelets in AD is presently not known, in view of the activity of ADAM-10 as an α-secretase, it is conceivable that the presence of lower levels of ADAM-10–mediated “non-amyloidogenic” α-secretase activity in peripheral cells such as circulating platelets could eventually contribute to amyloid neuropathogy in AD.

Based on this hypothesis, and on the presence of S100A7 in the serum (Pasinetti, unpublished observation), we initiated a series of studies to test the feasibility of using S100A7 as a means of promoting non-amyloidogenic” α-secretase activity as potential novel therapeutic approach in AD.

**Figure 9:**
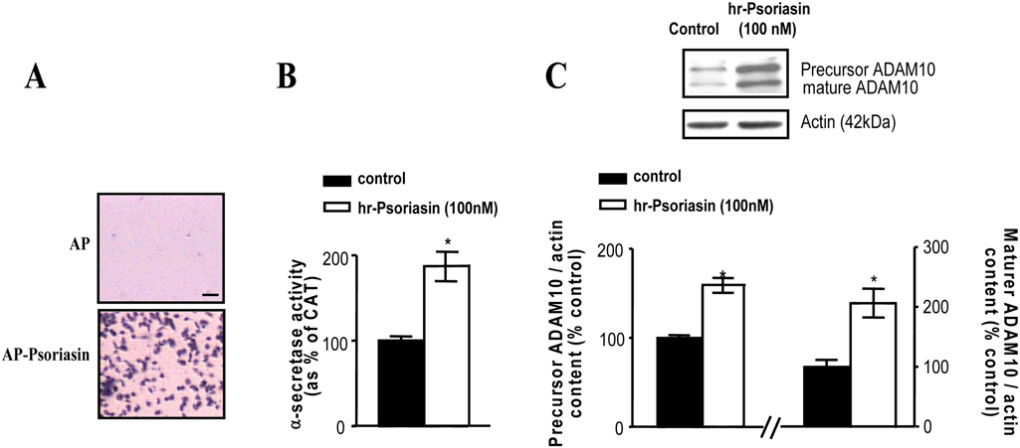
S100A7 receptor-binding activity in human platelets and modulation of α-secretase activity and ADAM-10 expression in platelet cells by S100A7 exposure. In this study, human platelet was prepared from blood collected from a cognitively normal control case. Cultured platelets were used for AP-S100A7 ligand-binding assay (*bottom*); parallel study using AP served as control staining (*A*). *B*, *C*, cultured platelets were treated with 100 nM h-S100A7 for 24 hours; parallel platelet cultures treated with vehicle served as controls. *B*, α -secretase activity in platelet lysates assessed using the α-secretase activity kit (R&D Systems). *C*, precursor and mature ADAM-10 content assessed by western blot analysis. *Inset*, representative western blot analysis of precursor and mature ADAM-10 in platelet lysates. *B*, *C*, values represent mean±SEM and are shown as percent of control cells; n = 3 independent cultures per group. *P<0.05, two-tailed Student *t*-test, hr-S100A7 vs. control group.

We first found that AP S100A7 binds intensely with purified human platelet cells, relative to AP control treated platelets (Fig. 9A). Thus, similar to neuronal cells, it is likely that platelet cells also express a cell-surface receptor. Based on this encouraging observation, we continued to explore a potential autocrine/paracrine role of S100A7 in modulating ADAM-10 in platelets through interaction with the S100A7 cell-surface receptor in platelets. We found that incubation of platelets with h-S100A7 (100 nM) leads to ∼two fold induction of “non-amyloidogenic” α-secretase activity (Fig. 9B) and elevated levels of ADAM-10 precursor as well as its mature catalytically active form (Fig. 9C).

Based on this evidence we initiated the generation of transgenic mice with constitutive overexpression of S100A7 in the brain to test the hypothesis that S100A7 may promote “non-amyloidogenic” α-secretase activity in the brain *in vivo*, and to test the potential therapeutic role of S100A7 expression in the brain on AD-type amyloid neuropathology.

### α-secretase activity increases in transgenic h-S100A7 mice brain

Based on the hypothesis that S100A7 might represent a potential promoter of “non-amyloidogenic” α-secretase activity in the brain precluding Aβ peptide generation we generated hS100A7 transgenic mice to test the hypothesis that S100A7 may ultimately influence AD-type neuropathology/neurodegeneration.

The expression of h-S100A7 transgene in these transgenic mice is driven by the cytomegalovirus immediate-early enhancer linked to the chicken β-actin promoter. h-S100A7 transgenic mice were generated and maintained using the C57B6/SJL stain background.

**Figure 10:**
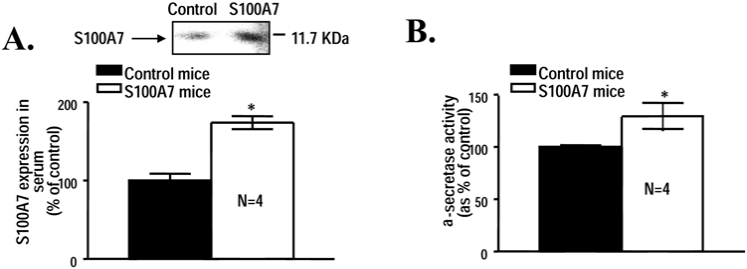
Elevated α-secretase activity in the brain of h-S100A7 transgenic mice. In this study, h-S100A7 transgenic mice were identified by dot blot hybridization of tail skin DNA samples with labeled h-S100A7 cDNA (for the h-S100A7 transgene). Expression of serum S100A7 (*A*) content in 1-month old h-S100A7 and age-, gender-, and stain-matched WT control mice was assessed by western blot analysis. The brains of h-S100A7 transgenic mice and WT control mice were assessed for α-secretase activity (*B*) using an activity assay kit (R&D Systems). Values represent mean±SEM and are expressed as percent of WT control mice group. *P<0.05, two-tailed Student t-test; n = 4 per group.

As expected, we found significant higher levels of immunodetectable S100A7, relative to age-, gender- and strain-matched wild-type (WT) control mice assessed by Western blot analysis (Fig. 10A). Moreover, we assessed for a potential role of hS100A7 expression in modulating α-secretase activity in our h-S100A7 mice. Excitingly, we found significant elevation of “non-amyloidogenic α-secretase activity in the brain of h-S100A7 transgenics compared to WT mice (Fig. 10B). The study suggest that therapeutic promotion of S100A7 expression in the brain may promote “non-amyloidogenic” α-secretase activity in the brain ultimately precluding generation of amyloidogenic Aβ peptides in the brain.

## Discussion

Diagnosis and monitoring of AD and the related dementias have long depended principally on clinical examination, especially cognitive testing. Establishment of biomarkers, which might assist in diagnosis or tracking of disease progression, would be a highly valuable addition to the care of patients. Such biomarkers are potentially available from body fluids and tissues as well as from brain imaging data. As specific disease-modifying therapies for Alzheimer's disease are developed, biomarkers may improve diagnostic accuracy and facilitate clinical trials, allowing a better gauge of treatment response [36].

Discovery-based proteomics discussed in this study has greatly enhanced our ability to reveal candidate proteins involved in various human diseases or biological settings. However, it is extremely challenging to validate these putative markers largely because of the enormous complexity of biological systems, heterogeneity of humans, and/or lack of high-throughput technology [37].

The identification of S100A7 as an AD biomarker could be a major advance in the understanding and treatment of AD, with a number of potential applications. Since S100A7 content is elevated in the CSF of AD dementia cases compared to neurological control cases, and these elevated S100A7 levels in the CSF selectively identify AD clinical severity, S100A7 could be a biomarker that quickly diagnoses AD before clinical signs have developed, allowing for early symptomatic treatment and the ability to chart the course of the disease accurately.

Our results show that S100A7 mRNA expression increases in the brain as a function of AD dementia and selectively correlated with AD amyloid plaque, but not neurofibrillary tangle, in a highly selective manner. This opens the question of whether S100A7 might be involved in amyloidogenesis. Our investigations suggest that it is, through activation of alpha-secretase activity. This S100A7-mediated anti-amyloidogenic activity coincided with the accumulation of both ADAM-10 precursor protein and the processed mature form. There was no detectable change in ADAM-9 and ADAM-17 (TACE) expression.

Aβ is thought to play a crucial role in the pathogenesis of AD. It is generally thought that the α-secretase pathway mitigates β-amyloid formation in the normal brain. The “non-amyloidogenic” α-secretase pathway starts with APP cleavage by α-secretase, which cuts within the β-amyloid domain and thus precludes β-amyloid generation. Several studies have suggested that α-secretases ADAM-9, ADAM-10, and ADAM-17, which belong to the ADAM (a disintegrin and metalloprotease) family of membrane-anchored cell-surface proteins, may be involved [38].

We do not know the mechanism by which the levels of ADAM-10 in circulating blood platelet cells of AD patients are reduced. But since ADAM-10 is an α-secretase, it is conceivable that these lower levels of anti-amyloidogenic α-secretase activity in peripheral cells could be caused by these reduced levels of ADAM-10. This could be part of the amyloid neuropathology in AD. Our finding showing >two fold promotion in “non-amyloidogenic” α-secretase activity and elevated levels of ADAM-10, following expression of exogenous hS100A7 to platelet cells supports this hypothesis.

β- or γ -secretase activity is not affected by human S100A7 expression in either CHO-APP_swe_ cells or in primary cortico-hippocampal neurons derived from Tg 2576 embryos. This in vitro evidence strongly suggests that the elevation of S100A7 expression in the brain of patients with AD might reflect a novel protective anti-amyloidogenic compensatory mechanism in response to the rampant AD brain amyloidogenesis. The mechanism by which S100A7 influences α-secretase and Aβ levels could involve p42/44 MAPK (Erk1/2) and PKC signaling. Our studies indicate that S100A7 increased Erk1/2 or PKC phosphorylation. Also, inhibitors of p42/44 MAPK or PKC have the effect of preventing h-S100A7 from influencing α-secretase activity.

Thus, it may be feasible to use S100A7 to promote anti-amyloidogenic α-secretase activity in peripheral cells. We tested this potential of S100A7 in promoting anti-amyloidogenic activity in the periphery and in the brain, *in vivo*, and the potential therapeutic impact on mitigating AD- type amyloid neuropathology. Our studies support the hypothesis that secreted S100A7, by interaction with cell surface receptor(s), can promote activation of ADAM-10, leading to induction of anti-amyloidogenic activity in neuronal cells.

We also note that the evidence suggesting that S100A7 associated induction in α-secretase mediated “non-amyloidogenic” processing might also be consistent with an alternate biological role of S100A7. For example, in view of the evidence suggesting that Aβ peptides might serve as a redox sensor and that oxidatively-induced Aβ may ultimately attenuate oxidative stress [39], the changes in S100A7 content in the CSF of AD brain might be also a reflection of oxidative stress changes besides Aβ content in AD. For example, there is evidence that oxidative stress *per se* may temporally precede increases in Aβ content in models of AD and that elevation in Aβ content might also function as negative feed-back signal on oxidative stress responses [40]. Thus, it might be possible that in the future changes in S100A7 content in AD might be also further explored to monitor oxidative stress changes associated to altered Aβ metabolism in AD [41].

Collectively, our experimental evidence tentatively suggests that S100A7 might be causally implicated in APP processing. While virtually nothing is known about S100A7 in AD pathogenesis, S1000A7 is a protein unrelated to other members of the S100 family [26], such as S100Bs and its specific role in the AD including potential modulation of immune-inflammatory responses besides APP processing discussed in this study, is presently unknown.

Our study provides unprecedented insight into the role of a novel biomarker of AD clinical dementia, and allow for the further systematic investigation of a potential novel therapeutic role for S100A7 via promotion of “non-amyloidogenic” α-secretase/ADAM-10 mediated responses and possibly through other mechanisms presently under investigation in our laboratory.
